# Emotional language processing in autism spectrum disorders: a systematic review

**DOI:** 10.3389/fnhum.2014.00991

**Published:** 2015-01-06

**Authors:** Alina Lartseva, Ton Dijkstra, Jan K. Buitelaar

**Affiliations:** ^1^Department of Cognitive Neuroscience, Donders Centre for Neuroscience, Radboud University Medical CentreNijmegen, Netherlands; ^2^International Max Planck Research School for Language Sciences, Max Planck Institute for PsycholinguisticsNijmegen, Netherlands; ^3^Donders Centre for Cognition, Radboud University NijmegenNijmegen, Netherlands

**Keywords:** autism spectrum disorders, asperger syndrome, social, emotion, affect, language

## Abstract

In his first description of Autism Spectrum Disorders (ASD), Kanner emphasized emotional impairments by characterizing children with ASD as indifferent to other people, self-absorbed, emotionally cold, distanced, and retracted. Thereafter, emotional impairments became regarded as part of the social impairments of ASD, and research mostly focused on understanding how individuals with ASD recognize visual expressions of emotions from faces and body postures. However, it still remains unclear how emotions are processed outside of the visual domain. This systematic review aims to fill this gap by focusing on impairments of emotional language processing in ASD. We systematically searched PubMed for papers published between 1990 and 2013 using standardized search terms. Studies show that people with ASD are able to correctly classify emotional language stimuli as emotionally positive or negative. However, processing of emotional language stimuli in ASD is associated with atypical patterns of attention and memory performance, as well as abnormal physiological and neural activity. Particularly, younger children with ASD have difficulties in acquiring and developing emotional concepts, and avoid using these in discourse. These emotional language impairments were not consistently associated with age, IQ, or level of development of language skills. We discuss how emotional language impairments fit with existing cognitive theories of ASD, such as central coherence, executive dysfunction, and weak Theory of Mind. We conclude that emotional impairments in ASD may be broader than just a mere consequence of social impairments, and should receive more attention in future research.

## Introduction

Autism spectrum disorder (ASD) is characterized by persistent impairments in social communication and interaction, restricted and repetitive behavior, and an onset of the disorder in early childhood (APA, 2013). While ASD was divided into subtypes in previous versions of the diagnostic and statistical manual of mental disorders (DSM), different forms of ASD are now combined in one broad category in the new DSM-5 (APA, 2013).

Clinically important diagnostic characteristics of ASD include lack of spontaneous seeking to share enjoyment, emotions, affect, interests, or achievements with other people, and a lack of emotional reciprocity. In his original description of autism, Kanner (1943) already emphasized the presence of emotional impairments, and characterized the patients as indifferent to other people, self-absorbed, emotionally cold, distanced, and retracted. However in the later years these emotional impairments were shifted to the background and were considered to be just a part of the core social deficits (Ritvo and Freeman, 1977; Rutter, 1978; Denckla, 1986; Fein et al., 1986). Most studies of emotion processing in ASD focused on facial emotion recognition (Jemel et al., 2006; Harms et al., 2010; Uljarevic and Hamilton, 2013). A recent meta-analysis concluded that facial emotion recognition is indeed impaired in the ASD population, although there is considerable variability between studies and the true effect size is probably much smaller due to publication bias (Uljarevic and Hamilton, 2013). However, the evidence from studies with facial stimuli alone is not sufficient to draw conclusions about the severity and extent of emotional impairments in ASD. The reason is that individuals with ASD have problems with processing faces in general, not restricted to only emotional faces (Jemel et al., 2006; Harms et al., 2010). In contrast to typical individuals, individuals with ASD do not spontaneously pay attention to faces, ignore the eye region and focus on less significant parts of the face (Jemel et al., 2006; Wolf et al., 2008; Senju and Johnson, 2009). So, even in studies that do report main group effects in facial emotion recognition, it is unclear whether the differences should be explained by a more specific emotional impairment, or by more general problems in face processing.

Comparatively little attention has been paid in research in ASD to processing of other types of emotional stimuli, such as emotional language (understanding of emotion-laden words, sentences and text; talking about emotions; perception and production of emotional intonation in speech). In fact, a lack of research on emotion processing in non-social tasks has been pointed out in earlier reviews (Gaigg, 2012; Nuske et al., 2013). If more generic emotional impairments exist in people with ASD that cause facial emotion processing deficits, we would expect to observe emotional impairments in language as well. The aim of this article is to provide a systematic review of the empirical literature with respect to emotional language in ASD, discuss the implications for our understanding of ASD, and give recommendations for future research.

In discussing emotional language in ASD it is important to keep in mind that many people with ASD have problems in language development and impairment in verbal and nonverbal communication. Phenotypically, most people with ASD have semantic, syntactic and pragmatic deficits, and a smaller number are known to have phonological deficits (Groen et al., 2008). On the most high-functioning end of the autism spectrum, people have no apparent language delay and normal to high verbal IQ with only minor difficulties with respect to pragmatic language use. In contrast, individuals on the most low-functioning end never develop any language at all. The majority of people with ASD range between these two extremes. This variation in verbal ability can be a major confound. Possible ways to deal with it include: (1) comparing high-functioning people with ASD to healthy controls matched on verbal IQ; (2) comparing people with ASD to other clinical groups with a similar level of language problems (for example, suffering from dyslexia or specific language impairment); and (3) including participants with a wide range of verbal abilities and testing if correcting for verbal IQ causes the effect of emotion to disappear.

## Aim

There is an evident lack of knowledge on how individuals with ASD process non-facial emotional stimuli, such as language. This paper aims to fill this gap by providing a systematic review of emotional impairments in ASD with a primary focus on emotional language. We will address the following questions:

1. Is there evidence for emotional impairments in the language domain? Is it present in different experimental paradigms? Does it depend on modality, such as visual (i.e., reading words and texts) or auditory channel (i.e., listening to words and sentences), and/or on complexity of information: simple (single words) vs. complex (sentences and discourse)? Does it affect primarily implicit measures (which the participant is not aware of or is not able to exert conscious control: difference in memory performance, error rates, physiological responses) or explicit processing of emotion (valence rating, verbal report)?
2. Does the degree of emotional language impairments in ASD individuals correlate with their clinical characteristics, such as the severity of cognitive and clinical linguistic impairments? If emotional impairments are caused by a linguistic or cognitive impairment, one would expect emotional impairments to be present more often and with greater severity in individuals with lower verbal or nonverbal IQ and a history of intellectual and language delay. In addition, IQ then would be negatively correlated with the severity of emotional impairments.
3. How do emotional language impairments fit within existing cognitive theories of ASD, such as the central coherence impairment, executive dysfunction, Theory of Mind account, and impairment of the mirror neuron system (MNS)? We will also explore whether the concept of alexithymia and motivational factors related to reward and punishment learning provide useful theoretical accounts of emotional language impairments in subjects with ASD.

## Methods

To identify relevant articles, we systematically searched PubMed and Google Scholar for papers published from 1980 onwards using the following search terms: #1 autism OR autistic OR Asperger OR ASD; #2 emotion* OR affect OR feeling; #3 #1 AND #2. We selected all original studies that compared the performance of a group of people diagnosed with ASD with controls on an emotion-related measure. Additionally, we manually searched the reference lists of these papers, and checked (using Google Scholar) all articles that were cited in the articles we selected.

Articles were included in our systematic review if they satisfied the following criteria:

– inclusion of participants with ASD diagnosed according to the official DSM or international classification of diseases (ICD) criteria;
– inclusion of at least one control group without ASD;
– the groups were compared with respect to their performance on an emotion-related measure, and either considered emotionally laden stimuli as an independent variable (such as emotional words, sentences or prosody) or studied the spontaneous production of emotionally loaded reactions (such as referring to one’s own or another person’s emotional state).

We excluded studies focusing on the processing of facial expressions of emotions, because these have been extensively reviewed elsewhere (Jemel et al., 2006; Harms et al., 2010), as well as intervention and drug studies. If the study measured multiple variables, performance on facial emotion recognition was not reviewed but other relevant outcomes were reported in the review. Thus, in contrast to other reviews, we focused exclusively on studies of relatively abstract emotional representations. At the end, we retrieved 33 studies with verbal/linguistic stimuli to be discussed in this review. A summary of all papers can be found in Table 1 including details on the ASD and control groups and the tasks administered. The studies retrieved vary with 8 reporting on emotional processing in children, 15 in adolescents, and 10 in adults with ASD. Nineteen/thirty three (57 percent) of the studies used formal research instruments autism diagnostic interview (ADI) or autism diagnostic observation schedule (ADOS) to confirm clinical diagnosis. Other diagnostic instruments included Autism Behavior Checklist (Volkmar et al., 1988), Asperger syndrome diagnostic scale (Goldstein, 2002), childhood autism rating scale (Schopler et al., 1980; Rellini et al., 2004), social communication questionnaire (Chandler et al., 2007), and others. Sample sizes for most studies were rather small (on average 18 participants in the ASD group, ranging from 6–37 participants) with only 12 (1/3) studies reporting about sample sizes larger than 20. Twenty-five studies included higher functioning subjects with ASD, whereas 8 studies investigated lower functioning participants. Seventeen studies did match ASD and control group on age and verbal/nonverbal IQ, but in 5 studies the groups significantly differed on IQ scores, 6 studies did not provide IQ scores for the control group, and 5 studies included multiple control groups partially matched on chronological age, mental age and IQ.

**Table 1:** **Studies of emotional language in autism**.

Authors	Groups characteristics: diagnosis, age, IQ	Stimuli and task	Reported results, *p*-values and effect size (Cohen’s *d*; if applicable)
*Lexicon and semantics*			
Hobson and Lee (1989)	21 LFA (Rutter 1974): Age: 18;09 (3.75) VIQ: 65.5 (16.6) 21 controls Age: 18;05 (3.9) VIQ: 66.5 (17.4)	Implicit: Matching words to pictures using Peabody Picture Vocabulary test	Implicit: + Subjects with ASD performed the test at the same level as the controls for abstract items and items with social content, but control subjects were significantly better on emotional items (between-group *t*-test: *t* = 2.79, *p* < 0.01, *d* = 0.44; group by type of item interaction: *F*_(1,20)_ = 5.89, *p* < 0.05).
Beversdorf et al. (1998)	10 ASD (ADI-R): Age: 30.8 (9.3) IQ: 109.7 (16.2) 13 controls Age: 30.6 (12.8) IQ: 117.3 (11.2)	Implicit: Listening to word lists, sentences and stories and subsequently recalling them.	Implicit: + Control subjects but not ASD recalled emotional sentences better than non-emotional sentences (significant group by emotion interaction: *F*_(1,21)_ = 7.394, *p* = 0.013, *d* = 0.47); no between-group differences in recall of word lists, sentences, coherent stories or sentences with theory of mind content.
Hillier and Allinson (2002)	10 LFA: Age: 12 VMA: 9.7, NVMA: 10 10 learning disability 10 TD controls (matched on MA) 10 TD controls (matched on CA)	Explicit: rate the level of embarrassment of a protagonist of a scenario.	Explicit: +/− Children with autism have difficulty with such concepts as empathic embarrassment (autism < MA-matched controls: *p* < 0.05; autism < CA-matched controls, *p* < 0.001) but showed a good understanding of other variables manipulated such as the presence of an audience.
Bauminger (2004)	16 autism (DSM-IV, ADI-R): Age: 11.14 (3) IQ: 92.81 (14.15) 17 controls Age: 11.5 (2.6) IQ: 98.35 (7.2)	Explicit: expression and understanding of jealousy	Explicit: +/− children with autism expressed jealousy in situations similar to their typical age mates but manifested it in different behaviors. Compared to TD children, children with autism were significantly less likely to look at the parent and/or the rival child (*F*_(1,29)_ = 4.10, *p* < 0.05, *d* = 0.75) but were significantly more likely to act toward them (*F*_(1,29)_ = 14.87, *p* < 0.001, *d* = 1.43). Moreover, children with autism revealed a less coherent understanding of the feeling.
Kennedy et al. (2006)	15 ASD (10 HFA, 3 AS, 2 PDDNOS; ADI, ADOS) Age: 25.49 ± 9.61 IQ: 96.1 (16.5) 14 TD controls Age: 26.07 ± 7.95 IQ: n/a	Implicit: counting Stroop task (count the number of words on the screen); surprise recognition test Stimuli: emotional (negative), neutral, and number words	Implicit; +/− Reaction time and accuracy: no effect of emotion, no emotion by group interaction Memory: effect of emotion in controls (*t*_(1, 8)_ = −4.02; *P* = 0.004), no effect of emotion in ASD group (*t*_(1, 11)_ = −1.15; *P* = 0.274), group by emotion interaction at trend (*F*_(1, 19)_ = 4.24, *P* = 0.062) fMRI: between-group difference in brain activity in PCC and PrC dorsal MPFC for (emotion vs. rest) contrast, in ventral MPFC / rACC for (emotion vs. neutral) contrast.
Rieffe et al. (2007)	22 ASD (DSM-IV): Age: 10.2 IQ: normal range 22 controls IQ: n/a	Explicit: Provide explanations about situations involving single and multiple emotions	Explicit: + children with autism have difficulties identifying their own emotions and less developed emotion concepts; they are more biased towards a single emotion perspective, especially within negative domain.
Corden et al. (2008)	17 AS (ADOS): Age: 34.2 IQ:112.9 17 controls Age: 32.3 IQ:109.9	Implicit: Attentional blink paradigm using emotional/neutral words (experiment 1) and neutral words of varying brightness (exp. 2); SCR response to emotion words. Explicit: subjective rating of arousal	Implicit: +/−, Explicit: − No differences in subjective ratings of arousal; no group * word type interaction in SCR measurement. In exp. 1, significant word type * time lag * group interaction (*F*_(3,96)_ = 5.2, *p* = 0.002). At shorter time lags, control subjects detected emotional words more successfully than AS group. In exp. 2, visual salience had the same effect on both groups (the main effect of group and all interactions involving group are nonsignificant)
Gaigg and Bowler (2008)	18 ASD (ADOS): Age: 32.8 IQ: 106.3 18 controls Age: 33.2 IQ:105.1	Implicit: Reading a list words containing unrelated neutral, semantically related neutral and emotional words, and recalling them at different time intervals.	Implicit: + ASD patients showed less correlation between SCR and subjective valence ratings (difference between groups: *t* = 2.62, d.f. = 34, *p* < 0.05) and higher forgetting rates for emotional words. In the typical group recall rates significantly decreased for unrelated neutral (*t* = 2.46, d.f. = 17, *p* < 0.05) and related neutral (*t* = 3.66, d.f. = 17, *p* < 0.005) words but not for the emotional words (*t* = 0.98, d.f. = 17, ns). For the ASD group, only the decrease in recall of arousing words over the 24 h period (*t* = 2.57, d.f. = 17, *p* < 0.05) was significant.
Mason et al. (2008)	18 ASD (ADI-R, ADOS-G, expert clinical opinion): Age: 26.5, IQ: 101.9 18 TD Age: 27.4, IQ: 105.5	Implicit: read short stories that contain physical, intentional or emotional inferences.	Implicit: + (neural activity) fMRI: The ASD group activated areas in right hemisphere which is interpreted as spillover processing.
South et al. (2008)	37 ASD (ADI-R, ADOS) Age: 19.7 (5.3) IQ: 107.7 (15.1) 38 TD controls Age: 19.2 (6) IQ:112.7 (14.1)	Implicit: visual search task; exposure task, word memory task and gambling task	Implicit: − No differences between groups on all 4 tasks. Emotion words memory subtest: group by word valence interaction: *F*_(2,70)_ = 0.14; *p* = 0.87; group by word arousal interaction: *F*_(2,70)_ = 0.33; *p* = 0.72.
Gaigg and Bowler (2009a)	25 ASD (ADOS): Age: 38.4 IQ: 105.2 25 controls Age: 36.2 (11.8) IQ: 105.8 (15.1)	Implicit: Attentional blink paradigm using emotional words, neutral words and male first names.	Implicit: + In the control group, detection rates of emotional words had significantly higher detection rate than names and neutral words (*F*_(2, 23)_ = 21.69, *p* = 0.001, *d* = 2.75). This was not the case for the ASD group (*F*_(2, 23)_ = 2.61, ns, *d* = 0.95). Emotionality effect was correlated with VIQ in ASD subjects (*r* = 0.502, *p* < 0.05) but not in controls.
Gaigg and Bowler (2009b)	22 ASD (ADOS): Age: 33.5 IQ: 98.7 22 controls Age: 35.2 (9.6) IQ: 103.7 (13.1)	Implicit: Memorizing a list of orthorgaphically related neutral and emotional words with subsequent recognition memory test.	Implicit: + Control subjects were significantly less likely to falsely remember emotional words (main effect of emotional/neutral word: *F*_(1,21)_ = 9.27; *p* < 0.01; effect size *r* = 0.55; *d* = 1.33). In subjects with ASD false recall rates of emotional and neutral words were not different (*F*_(1,21)_ = 0.49; ns; effect size *r* = 0.15; *d* = 0.31).
Williams and Happe (2010)	21 ASD (DSM IV-TR): Age: 12.3 VIQ: 73.24; PIQ: 67.10 21 controls (CA and MA matched)	Explicit: Report their own experience on various emotions and recognize them in video clips	Explicit: − No between-group difference in patterns of performance; in both groups social emotions were more difficult to recognise and report than non-social emotions. In ASD, verbal mental age was significantly correlated with quality of reports of both social (*r* = 0.66, *p* = 0.003) and non-social (*r* = 0.65, *p* = 0.004) emotion experiences. In contrast, in controls VMA was significantly correlated with the ability to recognise non-social emotions (*r* = 0.56, *p* = 0.008), but not social emotions (*r* = 0.32, *p* = 0.16).
Han et al. (2014)	10 ASD (ADOS) Age: 16.03 (1.9) IQ: 81.5 (8.9) 10 controls Age: 14.9 (2.7) IQ: 106 (10)	Explicit: detecting emotionally negative words	Explicit: + Participants with ASD made more errors detecting emotionally negative words (53.0 ± 14.6% correct) compared to control group (90.3 ± 9.1% correct) fMRI: increased activity in fusiform gyrus in ASD group in response to emotion words
*Voice-intonation*				
Boucher et al. (2000)	19 LFA (DSM-IV) Age: 9;7 VMA: 5;11, NVMA: 8;9 19 SLI (matched on CA and MA) 19 TD controls (matched on MA)	Explicit: Naming expressed emotion in a voice sample, matching voice to facial expression	Explicit: +/− On vocal affect naming ASD were better than SLI (*p* < 0.5, *d* = 0.4) and not different from controls. On matching vocal to facial expressions ASD were superior to the children with SLI (*p* < 0.01, *d* = 1.09), but impaired relative to TD children (*p* < 0.01, *d* = 1.03).
Lindner and Rosén (2006)	14 ASD (ASDS, PDD Checklist): Age: 10.21 (2.89) VIQ: 114.8 (17.15) 16 TD Age: 10.19 (3.12) VIQ: 117.3 (14.3)	Explicit: Naming emotions in photos, videos, intonation, verbal content and combined stimuli.	Explicit: + Participants with ASD were worse at correctly identifying emotion in prosody, *F*_(1,26)_ = 14.52, *P* < 0.01, partial eta squared = 0.36
Korpilahti et al. (2007)	14 AS (ADI-R, ADOS-G): Age: 11.2 (9–14) IQ: 110 (84–150) 12 fathers of AS: Age: 42.8 (37.5–49.5) 13 controls Age: 10.8 (9.1–11.7), IQ: n/a 12 fathers of controls	Implicit: Passive listening to words with neutral and angry intonation	Implicit: + In ASD group were found neural difficulties with processing of affective prosody, based on N1 and MMN evoked potentials. N1: longer latencies for children with ASD compared to controls (*p* = 0.021, *d* = 0.538), no difference for fathers. MMN: greater amplitudes of early MMN and shorter latencies of late MMN (*p* = 0.041) in children; significant differences in both MMN latency values (eMMN, *p* = 0.001; lMMN, *p* < 0.001) in fathers.
Peppé et al. (2007)	31 HFA (ICD-10, DSM – IV, CARS, Gilliam Autism Rating Scale, ADOS) Age: 9;10 (2.3) VMA: 7.09, VIQ: 81.6 (15.6) 72 TD children Age: 6;10 (1.5) VMA: 7.53, VIQ: 107.5 ( 9.6) 33 TD adults Age: 18–59	PEPS-C: a prosody comprehension and production task including affective prosody	Explicit: + ASD group performed worse both in perception (*F*_(1,97)_ = 16.21, *p* < 0.001, partial eta squared 0.14) and production (*F*_(1,97)_ = 11.32, *p* < 0.01, partial eta squared 0.10) of affective intonation
Järvinen-Pasley et al. (2008)	21 ASD (DSM-IV, ICD-10): Age: 12.55 (2.50) VIQ: 84 (19.33), NVIQ: 89 (14.44) 21 TD Age: 12.21 (2.15) VIQ: 87 (21.32), NVIQ: 88 (17.92)	Explicit: PEPS-C test: a prosody production and comprehension task including affective prosody	Explicit : + ASD group performed worse on Affect (Affective intonation) subtest: *t* = −2.38, *p* = 0.022
Volden and Sorenson (2009)	32 ASD (DSM-IIIR or DSM IV): Age: 10 (2.4) VMA: 7.9, NVMA: 10.6 29 controls Age: 8.8 (2.4) VMA: 8.9, NVMA: 10.4 26 controls Age: 8.8 (2.4) VMA:8.1, NVMA:8.7	Explicit: Production and perception of “bossy” and polite requests	Explicit: − participants with ASD were as adept as controls in both producing and judging polite ((*F*_(1, 85)_ = 0.607, n.s.) and bossy requests ((*F*_(1, 85)_ = 1.46, n.s.).
Grossman et al. (2010)	16 ASD (DSM-IV, ADI-R, ADOS): Age: 12;4 (2;3) IQ: 106.7 (10.6) 15 controls Age: 12;7 (3;1) IQ: 108.9 (11.3)	Explicit: classifying filtered and unfiltered spoken sentences as neutral, sad or happy	Explicit: − No differences between groups in affective prosody perception task: main effect of group: *F*_(1,29)_ = 0.44, *p* = 0.51, partial *η*^2^ = 0.02, group by task interaction: *F*_(1,29)_ = 0.35, *p* = 0.56,partial *η*^2^ = 0.01, group by emotion interaction: *F*_(1,29)_ = 2.3, *p* = 0.11, partial *η*^2^ = 0.07.
Hesling et al. (2010)	8 ASD (DSM-IV-R, ADI-R): Age: 23.38 (2.1) IQ: 89 (7.9) 8 controls Age: 23.05 (2.02) IQ: 128.3 (4.6)	Explicit: PEPS-C test: a prosody comprehension and production task including affective prosody. Implicit: fMRI task: listening to speech stimuli with varying intonation, rhythm, focus and affect prosodic aspects.	Implicit: +, explicit: + ASD subjects were significantly lower on all prosody tasks (*p* < 0.001); fMRI: ASD group showed differences in brain activation.
Kuchinke et al. (2011)	15 AS (DSM, ICD-10): Age: 35.6 (6.9) IQ: normal range 19 controls Age: 34.8 (7) VIQ: different NVIQ: matched	Implicit: Passive listening to sentences spoken with neutral or emotional intonation, pupil size recording Explicit: subsequent valence rating.	Implicit: +, explicit: + Pupil diameter measurement: In passive listening condition, there was a significant emotion by group interaction (*F*_(2,64)_ = 4.634; *p* < 0.05). AS group demonstrated increased pupillary response to negative sentences and decreased response to positive sentences, compared to controls. During explicit evaluation, no interaction or main effect involving group was significant. The main effect of emotion was significant (*F*_(2,64)_ = 10.236; *p* < 0.001, greater pupil dilation in response to emotional sentences) Subjective valence ratings: Lower ratings of positive (*t*_(1,24)_ = 5.643; *p* < 0.001) as well as negative sentences (*t*_(1,24)_ = −2.206; *p* < 0.05) sentences in the AS group compared to control group.
Eigsti et al. (2012)	16 ASD (DSM-IV, ADI-R, ADOS-G): Age: 13.7 (2.8) IQ: 96.7 (14.9) 11 TD Age: 13.7 (2.6) IQ: 111.9 (10.9)	Implicit: Listening to angry and neutral sentences in an fMRI scanner	Implicit: + (neural activity) fMRI: in TD group compared to ASD, angry sentences elicited more activity in the L IFG and lower activity in the R MFG and R STG
*Production*			
Tager-Flusberg (1992)	6 ASD (Rutter, 1978, DSM-III-R): Age: 3–8 IQ: 61–108 6 Down syndrome (matched on CA and language ability)	Implicit: Analysis of spontaneous speech samples collected over the course of 1–2 years	Implicit: + autistic children were comparable to the Down syndrome control subjects in their talk about desire, perception and emotion. However, they used significantly less language to call for attention (*t*_(10)_ = 4.47, *p* < 0.001) and to refer to psychological states (*t*_(10)_ = 1.98, *p* < 0.05)
Capps et al. (2000)	13 LF A (DSM-III-R, CARS, ABC) Age: 12.6 IQ: 75.2 13 TD controls age: 6.0 (1.6) 13 DD controls Age: 9.8 (2.8) IQ: 78.9 (13.1)	Implicit: Telling a story based on a picture book	Implicit: + Compared to TD, ASD and DD children produced shorter stories (*p* < 0.05, *d* = 1.05), less complex syntax (*p* < 0.02, *d* = 1.04), more restricted range of evaluative forms (*p* < 0.1, *d* = 1.14); used causal attribution less often in the references of the emotional state (*p* < 0.005, *d* = 1.44), but more often to describe physical events (*p* = 0.07, *d* = 0.79). No difference between ASD and DD groups was found. In ASD, but not in DD group, there were significant correlations between theory of mind performance and narrative qualities (*t*_(10)_ = 0.56 − 0.78, *p* < 0.05); and conversational competence with syntactic diversity (*t*_(10)_ = 0.67, *p* < 0.05) and evaluative diversity (*t*_(10)_ = 0.75, *p* < 0.01)
Adams et al. (2002)	19 Asperger syndrome (ICD-10): Age: 13.81 (2.6) IQ: 92.53 (22.8, 71–141) 19 conduct disorder Age: 14.5 (1.6) IQ: 85.5 (13.2)	Implicit: Two structured conversations based on ADOS: on a social-emotional topic and about a non-routine event.	Implicit: + In social-emotional conversation the AS group showed more response problems (*p* < 0.0005, *d* = 1.97); more pragmatic problems (*p* < 0.0005, *d* = 2.78); same in the non-routine conversation: for overall response problems *p* = 0.006, *d* = 1.49; for pragmatic problems *p* = 0.008, *d* = 0.7.
Pearlman-Avnion and Eviatar (2002)	13 ASD (DSM-IV) Age: 11.54 (8–16) 13 younger TD Age: 7.36 13 older TD Age: 11.5 13 WS Age: 14.39 (8–21)	Implicit: tell a story after a slide show	Implicit: + Affective expression analysis: HFA group performed significantly lower than TD and WS group. Interaction between Group (ASD, Williams syndrome, typical) and task (emotional, informational): *F*_(3, 48)_ = 3.85, *p* < 0.05.
Müller and Schuler (2006)	13 ASD (ADI, ABC): Age: 8–11 IQ: 70–140 13 TD controls Age: 8–11 IQ: n/a	Implicit: Spontaneous interactions of subjects with family members.	Implicit: + Children with AS and HFA engaged in a higher proportion of affect marking and provided a higher proportion of affective explanations than typically developing children (*p* < 0.01, *d* = 0.68), yet were less likely to initiate affect marking sequences (*t* = −2.75, *p* < 0.01, *d* = −0.55) or talk about the affective responses of others (*t* = 1.52, *p* < 0.05, *d* = 0.65). No significant differences in terms of the marking of positive and negative affect.
Losh and Capps (2006)	28 ASD (DSM, ICD-10, ADI-R): Age: 11.1 (8–13) IQ: 98 (76–132) 22 TD controls Age: 10.6 (8–12) IQ:108 (102–123)	Implicit: Discourse analysis of personal accounts of emotional and non-emotional events	Implicit: + No difference in describing non-emotional events between the groups. The descriptions of emotional events in ASD children were less contextually appropriate, less elaborate and contained less evaluations of causes, especially for complex emotions (effect sizes 0.81–1.52, *p* < 0.05) and self-conscious emotions (effect sizes 0.77–1.74, *p* < 0.05).
Bang et al. (2012)	20 HFA (DSM-IV, ADOS-3, SCQ): Age: 11;0 (1;11) PIQ: 106.5 (17) 17 TD Age: 10;10 (1;5) PIQ: 112.5 (14)	Implicit: free conversation with experimenter	Implicit: − No difference between groups in terms of producing emotion and desire terms (ASD group: 3.40 (0–12 words), median 2.5; typical group: on average 3.76 (0–13 words), median 2; Mann–Whitney *U* = 181.50, *p* = 0.72, *r* = 0.06)
Brown et al. (2012)	30 ASD (DSM-IV): Age: 6–14 IQ: 107.5 (15.6) 20 TD Age: 8.95 (2.35) IQ: 111.00 (11.53)	Explicit and Implicit: Autobiographical memory interview on 3 topics: earliest memory; (2) positive; and (3) negative memory	Explicit: −, implicit: + No difference in response rate and narrative length between different types of narratives. Usage of internal state terms: Typically-developing children used more emotional terms (*F*_(1, 126)_ = 9.11, *p* < 0.01, *n*2 = 0.07).
Siller et al. (2014)	21 ASD (ADOS) Age: 7.2 (1.5) VIQ: 100.6 (16.3) 24 TD Age: 6.8 (1.6) VIQ: 99.4 (10.9)	Implicit:telling a story after a picture book	Implicit: + ASD group produced shorter narratives (*F*_(1,40)_ = 13.6, *p* < 0.01), less references to characters emotions (*F*_(1,40)_ = 13.2, *p* < 0.01) but the same amount of references to cognitive states (*F*_(1,40)_ = 3.4, *p* = 0.07 (*ηp2* ≥ 0.08)).

## Is there evidence for emotional impairment in emotional language?

First, we addressed the question of whether abnormalities in emotional language processing are specific to particular cognitive domains, or are more general, i.e., independent of the type of information involved. In the next section, we group all articles in terms of the cognitive task used.

### Comparison of different tasks

Emotional language has been investigated in a variety of tasks including recognition and recall, stimulus detection, and discourse and reasoning.

Typically developing people remember emotion laden words and sentences better than neutral stimuli (Dolcos et al., 2004). Some reports indicate that this is not the case for subjects with ASD. In one study, participants listened to and had to memorize neutral and emotional sentences, among other conditions (Beversdorf et al., 1998). Memory performance did not differ between ASD and control participants on scrambled word lists, neutral sentences, sentences which made a coherent story together with other sentences from the list, and sentences describing other people’s mental states and perspectives. The only difference between the two groups was on recall of emotional sentences where typical participants showed better performance compared to other conditions, while ASD subjects did not (Beversdorf et al., 1998). A similar finding was obtained in studies with visually presented single words (Kennedy et al., 2006; Gaigg and Bowler, 2008, 2009b). Control participants showed better recognition memory for emotional words compared to neutral words, had better recall rates for emotion words over a 24 h period, and were less likely to falsely recall them in an illusory memory paradigm. In ASD subjects, however, those effects were significantly diminished. The current view is that typical people remember emotional stimuli better because they perceive these stimuli as potentially motivationally significant and allocate additional processing resources. This process is subserved by medial temporal lobe structures and results in better encoding and retrieval (Dolcos et al., 2004). Lack of emotional memory effect in the ASD participants can mean that they either did not perceive the emotional words or sentences as more salient and motivationally relevant, or failed to efficiently encode them because of an impairment at a later processing stage. In contrast, another study did not find a difference in memory performance for emotion words between ASD and typical participants (South et al., 2008). In this study participants read word lists and were then immediately tested for recognition memory. This result suggests that the differences between the ASD and typical groups on emotional memory could be dependent on time and could be less prominent at immediate recall/recognition.

Subjects with ASD also display abnormalities in their automatic attentional reaction to emotionally salient stimuli. In an attentional blink paradigm, subjects were asked to read words in a rapid serial visual presentation (RSVP) stream and detect target words printed in a red color (Gaigg and Bowler, 2009a). Normally, the probability of successfully detecting a target word depends on the time interval between the presentation of the preceding target word (T1) and the current target word (T2). If the T1 was presented just before the T2, then at the time the T2 is presented most of the attention resources are still engaged in processing the T1, and therefore the probability of successfully detecting T2 significantly drops (“attentional blink” effect). However, this attentional blink phenomenon does not occur with emotional words, which are detected with high accuracy irrespective of the timing. This emotional modulation of attentional blink is thought to be a result of emotion words being perceived as more salient and motivationally relevant and thus receiving more attentional resources, primarily because if the involvement of the amygdala in detecting of emotional words (Anderson and Phelps, 2001). In two studies, the predicted modulation of attentional blink by T2 valence was found in controls, but not in ASD. In the ASD group, the detection rates of neutral and emotional T2s were not statistically different (Corden et al., 2008; Gaigg and Bowler, 2009a). This result suggests that individuals with ASD do not perceive emotional words as more salient, suggesting an impairment in the amygdala functioning or in the connectivity between the amygdala, cingulate cortex and frontal cortical areas in the ASD group (Gaigg and Bowler, 2009a).

Studies of reasoning about emotions expressed in discourse also showed differences between ASD and control individuals. One study investigated children’s responses in a structured conversation. This study found that children with Asperger syndrome more frequently gave inadequate or no response when talking about an emotional topic compared to a neutral one (Adams et al., 2002). Two more studies investigated emotion word use during telling a story about a fictional or real event. They found that children with ASD talked significantly less about emotions, desires and beliefs compared to typically developing control children. Children with ASD also less frequently referred to emotional states as a cause of one’s action (compared to TD group), although both groups talked equally often about causes for physical events (Capps et al., 2000; Losh and Capps, 2006). Similar results were obtained in a study which asked adult participants to watch a film clip displaying interactions between people. Adults with ASD were less likely than controls to include the characters’ emotional state in the description or explanation of their behavior (Barnes et al., 2009). Overall these findings show that individuals with ASD have problems with processing and handling emotional language in memory paradigms, automatic information processing, and in discourse and reasoning.

### Comprehension vs. production tasks

There is debate on whether or not subjects with ASD have abnormal profiles of production and comprehension of language. Some studies find better production than comprehension in children with ASD for language in general (Hudry et al., 2010b; Miniscalco et al., 2012) while other studies report a more uniform profile of language development (Jarrold et al., 1997). Three of the studies reviewed here used the PEPS-C prosody test which consists of perception and production subtests, including affective prosody; all of these studies showed deficits in both perception and production subtests (Peppé et al., 2007; Järvinen-Pasley et al., 2008; Hesling et al., 2010). In spontaneous dialog or interview, participants from the ASD group were less likely to mention emotions, describe emotional states (Tager-Flusberg, 1992; Adams et al., 2002; Müller and Schuler, 2006), or use emotional state for a causal explanation of characters’ actions (Capps et al., 2000; Pearlman-Avnion and Eviatar, 2002; Brown et al., 2012). In some studies, this neglect was specific to emotions and not present when talking about other types of events (Losh and Capps, 2006); however, one study found no group differences between ASD and typically developing children (Bang et al., 2012).

In all, the data suggest that emotion language impairments are present in both production and comprehension modes. There is no evidence for an imbalance between production and comprehension emotion language skills in ASD.

### Modality: visual vs. auditory

Auditorily presented speech unfolds over time, so participants need to continuously pay attention; it is also not possible to go back and listen to parts of it again if something was missed or misheard. Analysis of auditory stimuli also starts earlier, with semantic analysis being made on the basis of partial information before the presentation of the word ends, which results in an earlier onset of semantic ERP effects (Holcomb and Neville, 1990). Previous studies have reported that auditory stimuli compared to visual also elicit stronger behavioral effects: larger and more robust effects of semantic priming and semantic expectancy on reaction times and accuracy (Holcomb and Neville, 1990; Holcomb et al., 1992), as well as greater amplitude and earlier onset of electrophysiological brain responses (Holcomb et al., 1992; Niznikiewicz et al., 1997). This means that visual tasks can be potentially less sensitive and require larger samples to have sufficient power to detect a difference between ASD and control groups. Processing of auditory stimuli compared to visual is also affected by timing of word presentation to a much greater degree (Anderson and Holcomb, 1995), and follows a different developmental trajectory (Holcomb et al., 1992).

From the studies that investigated perception of emotional language, 8 studies presented the stimuli visually (Kennedy et al., 2006; Corden et al., 2008; Gaigg and Bowler, 2008; Mason et al., 2008; South et al., 2008; Gaigg and Bowler, 2009a, b; Han et al., 2014) and 11 studies auditorily (Beversdorf et al., 1998; Boucher et al., 2000; Lindner and Rosén, 2006; Korpilahti et al., 2007; Peppé et al., 2007; Järvinen-Pasley et al., 2008; Volden and Sorenson, 2009; Grossman et al., 2010; Hesling et al., 2010; Kuchinke et al., 2011; Eigsti et al., 2012). In the 11 studies that used auditory modality, 8 studies used explicit measures (participants were asked to identify the expressed emotion), and only one study investigated implicit behavioral measures (memory performance, Beversdorf et al., 1998). In contrast, 7 out of 8 studies using visually presented stimuli looked at implicit measures: reaction time, stimulus detection rate, memory performance, while only three used explicit measures: rating emotional arousal of words on a scale (Corden et al., 2008; Gaigg and Bowler, 2008) or detecting emotionally negative words among neutral (Han et al., 2014). The fact that visual and auditory stimuli were used in different paradigms makes it difficult to assess whether the modality in which the stimulus is presented makes a difference. Explicit measures in studies with auditorily presented stimuli were more likely to show a group difference compared to studies with visual stimuli, suggesting that the transient nature of the auditory signal may indeed make the task more difficult for the ASD group. However, because of the small number of studies we can’t draw a definite conclusion. Future studies could address this issue by using visual and auditory language stimuli in the same participants and the same task.

### Complexity of information: words vs. sentences

Understanding single words for people with ASD is less problematic than understanding complete sentences and natural speech (Tager-Flusberg et al., 2005; Williams et al., 2008).

As indicated in the previous section, many studies on the processing of emotion language have focused on discourse, such as talking about emotional topics and making inferences about emotional states (Adams et al., 2002; Losh and Capps, 2006; Barnes et al., 2009). An alternative explanation could be that the individuals with autism have difficulties expressing complex thoughts in general and that problems in reasoning on emotional topics are not very emotion specific. Therefore, it is important to note that several studies also found impaired performance in the ASD group on tasks that involved detecting or remembering single emotional words. The ASD group performed significantly worse than non-ASD subjects specifically on memory for emotional words (Kennedy et al., 2006; Gaigg and Bowler, 2008, 2009b), although the overall memory performance was similar for ASD and non-ASD subjects. Participants with ASD had less understanding of emotion-related words, but this was not the case for abstract non-emotional words (Hobson and Lee, 1989). In another study where participants had to memorize sentences, participants with ASD showed impaired performance on emotional sentences, but were just as good as controls in remembering neutral sentences with social and Theory of Mind content (Beversdorf et al., 1998). Thus, it was not the case that emotional sentences were more difficult for subjects with ASD because of their relation to social context with which ASD participants would be less familiar. In all, it is unlikely that the deficient performance in processing emotional verbal stimuli in the studies reviewed above is due to a general effect of stimulus complexity.

### Explicit vs. implicit measures

Some studies used explicit emotion measures to investigate emotion processing in ASD: in those studies participants were asked to assign the stimuli to a particular category (happy or sad) or to rate it as positive or negative, or as more or less emotionally arousing. Other studies applied indirect measures of emotional processing, such as the effect of emotion on attention or memory, physiological responses, brain activity, and spontaneous talk about emotion.

Indirect and implicit measures are problematic, because the absence of an emotion effect could be due to a deficit outside the emotional domain, for example, in attention or motivation. Thus, the absence of an emotion effect in the ASD group should be interpreted with caution. On the other hand, explicit measures can also be problematic, especially in subjects with high IQ. Even though these individuals may have impairments in emotion processing, they can use their analytic abilities to design alternative strategies to label stimuli as positive or negative. For example, emotional intonation differs from neutral on a number of acoustic characteristics such as pitch height and variation (Bänziger and Scherer, 2005). If individuals with ASD would focus on isolated features in a prosody perception task, they would be able to correctly classify stimuli as emotional or neutral, but the way they process the stimuli still may be fundamentally different from typical controls, who use a more holistic strategy. Similarly, in facial emotion recognition studies, distinguishing between positive and negative emotion can be done based on single feature, such as upturned or downturned mouth corners, which fits with some of the studies finding atypical gaze patterns in ASD (Neumann et al., 2006). Thus, rather normal explicit responses in the ASD group can only be interpreted in combination with other measures.

Approximately half of the studies using explicit measures report differences between ASD and controls. It is important to consider the difficulty of the applied task. For instance, when participants were asked to classify the auditorily presented sentences as sad and happy, both groups performed in a similar way (Grossman et al., 2010). In more difficult tasks, on the other hand, participants with ASD made more errors than controls: for example, when they had to choose between four response options in a task that required identifying emotion in intonation, (Lindner and Rosén, 2006), or when they had to identify complex emotions of social nature such as embarrassment or jealousy (Hillier and Allinson, 2002; Bauminger, 2004; Rieffe et al., 2007). However, some studies also found lower performance in the ASD group in a simple 2-alternative choice task between sad and happy intonation (Peppé et al., 2007; Hesling et al., 2010), suggesting that the task difficulty should be considered in combination with age and functioning level of the participants. To conclude, in certain circumstances participants with ASD have trouble with fine differentiation between various emotions and perform worse than control subjects. Unfortunately, all of the studies either provided participants with response options, or asked to rate them on one single scale. Potentially, naming the emotion in a free response task, or rating the stimuli on several scales at once (for example, valence and arousal) and having to distinguish between these different aspects, would be more challenging, and this kind of task should be more likely to uncover group differences.

Studies that use implicit measures report strongly variable findings. Behavioral results are very much dependent on the task used, and with respect to some tasks, the results are still preliminary. For example, only 2 studies so far report attenuated attention blink effect (Corden et al., 2008; Gaigg and Bowler, 2009a), and 4 studies find a weaker effect of emotion on memory in ASD compared to non-ASD subjects (Beversdorf et al., 1998; Kennedy et al., 2006; Gaigg and Bowler, 2008, 2009b) but another study does not (South et al., 2008).

With respect to activity of the autonomic nervous system, the studies measuring skin conductance response (SCR) modulation found typical responses during reading emotion words compared to neutral ones in the ASD group (Corden et al., 2008; Gaigg and Bowler, 2008). These studies used highly arousing emotional words, such as profanities, taboo words and sexually explicit words, and the highly arousing nature of these stimuli could be the reason why they elicited an autonomic response not only in typical but also in ASD group. Another study measuring pupillary response to the stimuli with an intermediate level of emotional arousal (sentences spoken with positive and negative intonation) showed increased response to negative stimuli and decreased response to positive (Kuchinke et al., 2011). This is in agreement with yet other studies showing absent or reversed responses of the autonomous nervous system in an anxiety-provoking situation in people with ASD (Kushki et al., 2013). Due to the small number of studies and differences in autonomic measures and design, it is not yet possible to reach a conclusion with respect to physiological correlates of emotions in ASD, but the preliminary evidence suggests that participants with ASD may be less sensitive to subtle variations in emotional valence, while stimuli with high emotional arousal are equally likely to elicit an autonomic response in ASD and typical population. More research in this field is needed.

Moving now to the studies of brain activity, our selection included 5 fMRI studies and one EEG study (Korpilahti et al., 2007). All of them report significant between-group differences with respect to processing of emotion language at the neural level. Studies report less deactivation of the default-mode network regions for visual or auditory emotional stimuli compared to neutral stimuli or rest in the ASD group (Kennedy et al., 2006; Hesling et al., 2010), suggesting that individuals with ASD may have trouble suppressing task-irrelevant regions and focusing on the relevant stimuli characteristics. The medial prefrontal cortex (MPFC) was more active during rest than during task performance in the typical group, and during task it was more active in response to emotional compared to neutral words (Kennedy et al., 2006). However, the ASD group failed to show any modulation of activity in this region in either of the comparisons. Medial prefrontal regions have been previously implicated in evaluation of stimuli as pleasant or unpleasant (Maddock et al., 2003) and in silent generation of emotionally positive or negative words (Cato et al., 2004). A lack of modulation of activity in this area in the ASD group suggests an impairment in the processes of evaluation, but it also indicates that people with ASD may engage in different kinds of cognitive processing during resting state, as indicated by higher activity in MPFC at rest in typical group compared to ASD group. Participants with ASD also showed greater activation in the left supramarginal gyrus (implicated in phonological processing) during an auditory task (Hesling et al., 2010), suggesting that the ASD group focused more on phonological rather than conceptual features of the input. Furthermore, studies with verbal stimuli find more activity outside of the language network in the ASD group: fusiform gyrus (Han et al., 2014), right hemisphere homologs (Mason et al., 2008; Eigsti et al., 2012), and brain areas typically involved in memory and control, such as parahippocampal gyrus and globus pallidus (Eigsti et al., 2012). These results suggest that individuals with ASD recruit a different network of brain areas during emotional language tasks, which results in less efficient processing and requires additional involvement of the homologous regions from the right hemisphere. The EEG study comparing electrophysiological responses to auditorily presented neutral and angry sentences found increased mismatch negativity (MMN) amplitude in the ASD group compared to the control group, suggesting that participants with ASD focused more on the low-level acoustic characteristics of the utterances, in contrast to typical group which was processing the stimuli in a more global way. Additionally, there were differences between group in terms of lateralization of the components. Typical children showed larger N1 amplitudes over the right hemisphere and larger late MMN amplitudes over the left hemisphere, but the ASD group showed less lateralization for MMN and N1, with greatest differences between groups over the right hemisphere (Korpilahti et al., 2007). However, this study did not have a behavioral measure, thus it is difficult to make direct inferences from differences in electrophysiological responses to the corresponding cognitive processes.

In sum, the studies reviewed here demonstrate that subjects with ASD have widespread impairments in processing emotional language, and these impairments are present in tasks tapping into different cognitive domains, in comprehension as well as production tasks, and tasks of varying levels of complexity. These impairments are also reflected in abnormal concomitant physiological and neural responses.

### Summary

To summarize, individuals with ASD are able to correctly identify words, sentences or stories as emotionally positive or negative (Volden and Sorenson, 2009; Grossman et al., 2010; Brown et al., 2012), but have difficulty with providing an in-depth explanation (Bauminger, 2004; Rieffe et al., 2007). Implicit behavioral measures show more robust and consistent between-group differences (compared to studies that use explicit behavioral measures). The overall tendency is that subjects with ASD have a poorer memory for emotional events (Gaigg and Bowler, 2008, 2009b), are less inclined to direct attentional resources to emotional stimuli (Korpilahti et al., 2007; Corden et al., 2008; Gaigg and Bowler, 2009a) and mention emotions in spontaneous conversations (Capps et al., 2000; Barnes et al., 2009) than typically developing participants. Studies of autonomic activity are very few in number and show mixed results. In contrast, neuroimaging studies report consistently diminished brain activation in response to emotional stimuli in subjects with ASD compared to typical participants. At least in studies using auditory stimuli, the brain activity results suggest that participants with ASD are more likely to focus on phonological features of the input rather than process it in the holistic manner, as we hypothesized in Section Explicit vs. Implicit Measures (Korpilahti et al., 2007; Hesling et al., 2010). However, only a small number of studies investigated brain activity (1 study with EEG and 5 studies with fMRI), therefore more studies are required before we can draw more firm conclusions.

It was shown that high-functioning subjects with ASD are able to interpret others’ emotional behavior correctly and react with an appropriate emotional response if they are provided with explicit cues, in spite of their problems with spontaneous emotional interactions (Begeer et al., 2008). There are two alternative explanations for the discrepancy between absent spontaneously applied and relatively intact cue-related emotional skills and responses in ASD. One option is that individuals with ASD have adequate and typical conceptual representations of emotions, but have trouble applying them in real life. The alternative explanation is that the emotion concepts of ASD individuals are actually different from typical individuals and are more similar to neutral concepts. The evidence from the behavioral studies of attention and memory (Beversdorf et al., 1998; Corden et al., 2008; Gaigg and Bowler, 2008, 2009a,b) points towards the latter alternative, and the few available neuroimaging studies suggest that individuals with ASD largely recruit the same neural networks for processing emotional and neutral language (Kennedy et al., 2006; Hesling et al., 2010). However, this is only a preliminary conclusion that needs to be backed up by more research, particularly on autonomic activity and electrophysiology.

## Emotional language impairment and general intelligence

About half to two-third of people with ASD also have some form of intellectual disability (Matson and Shoemaker, 2009). Language impairment in ASD has a more complex pattern. Higher order linguistic functions, such as semantics and pragmatics (understanding contextual and non-literal meaning, inferring speaker intentions) are almost universally impaired in ASD, while phonology, lexicon and syntax are affected to varying degrees in different subgroups (Groen et al., 2008; Eigsti et al., 2011). Some researchers also report more deficits in language comprehension than in production (Hudry et al., 2010a). In the following part we will discuss the link between impairment in emotional language processing and (1) history of language delay; (2) presence of intellectual disability; and (3) individual differences in verbal and nonverbal IQ within normal range.

### Are impairments in emotional language related to language delay?

By the definition of the DSM-IV criteria, subjects with Asperger have normal milestones for language acquisition in the first years after birth (APA, 2000). If emotional impairments in ASD are in part due to a delay in language acquisition, we would expect that studies including subjects with Asperger syndrome would more often report negative results, in other words find less of even no differences between the control and patient group on emotional language measures. However, this appears not to be the case. Individuals with Asperger syndrome have serious problems in talking about social-emotional topics (Adams et al., 2002). Furthermore, they have an abnormal Event-Related Potentials (ERP) pattern when hearing angry intonation (Korpilahti et al., 2007), fail to show modulation of attention blink effect by emotional content of the words (Corden et al., 2008), and show a different pattern of pupillary response when hearing emotional sentences (Kuchinke et al., 2011). In all these aspects of emotion processing, subjects with Asperger syndrome differ from controls, despite their rather intact early language skills and absence of language delay. Therefore, their impairment in emotional language cannot be explained by developmental delay in language and communication. However, none of the studies compared a group of participants with Asperger syndrome to a group with autism and language delay within the same paradigm. Therefore, the question remains whether individuals with classic autism who frequently have early language delays, would demonstrate a greater degree of emotion language impairment under the same task conditions.

If the overall level of language development is a decisive factor for the normal development of emotional competence and processing of emotional stimuli, emotional language impairments should also diminish or even disappear when people with ASD catch up in their language development with their peers. However, this does not appear to be the case, since the emotional impairments do not disappear when the participants become older and acquire better language skills. The participants in the studies ranged from 3-year-old children to adolescents and adults, and differences in emotional language processing occurred in subjects with ASD in all age groups. Furthermore, although high-functioning individuals with ASD, and thus without language delay, from various age groups were able to provide adequate explicit evaluations of emotional stimuli, they showed atypical performance compared to control subjects on measures of memory performance, attention, and autonomic activity in relation to the processing of emotional language stimuli. The cause of the absence of behavioral differences in some tasks remains an open question, but one likely explanation is that high-functioning ASD subjects develop compensatory strategies by explicitly learning the relation between a situation and an emotional label (Begeer et al., 2008; see also Hobson, 1991).

Comparison of the results to a non-emotional task of similar difficulty can also help to address the issue of whether or not emotional language difficulties in ASD are a consequence of more general language delay. Several studies have used such control tasks. They found that the atypical performance in the ASD participants was relatively specific to emotional stimuli, and did not extend to other semantic features of the verbal materials: namely semantic relatedness (Gaigg and Bowler, 2008), distinctiveness of individual words (Corden et al., 2008; Gaigg and Bowler, 2009a), or the conceptual content of sentences (Beversdorf et al., 1998).

### Are impairments in emotional language related to the presence of intellectual disability?

The next question is whether the emotional impairments are related to the presence of general intellectual disability. If so, emotional impairments should show a different degree of severity in individuals with ASD with high and low IQ. One possibility is that individuals with ASD and intellectual disability would show a greater degree of emotional impairment. Most of the studies we identified tested individuals with normal or above normal intelligence, and only 8 studies included a sample of low-functioning subjects (Hobson and Lee, 1989; Boucher et al., 2000; Capps et al., 2000; Hillier and Allinson, 2002; Pearlman-Avnion and Eviatar, 2002; Williams and Happé, 2010). It is difficult and even problematic to estimate whether and how emotional language stimuli are processed in low-functioning subjects with ASD, because more complex tasks can’t be administered in this population. Further, the problems specific to emotional information add up with problems in understanding the instruction and performing the task in general. Thus, in more difficult tasks specific difficulties with emotion processing may be obscured, for instance, by the effects of shorter attention span, poor understanding of the task, tendency for impulsivity, or response perseveration. Simpler tasks, on the other hand, may lead to ceiling effects in both groups.

Notwithstanding these methodological problems, some studies reported that low-functioning individuals with ASD performed worse on emotion language task than mental age-matched healthy controls (Rieffe et al., 2007). In contrast, other studies found that low functioning ASD subjects performed equally (Capps et al., 2000; Williams and Happé, 2010) or even better (Boucher et al., 2000) than children with a linguistic or intellectual impairment. These discrepant findings could reflect differences in emotional skills between the groups, but could also be due to the task demands being too high, particularly for the children with an intellectual disability. This confound makes it difficult to determine whether the emotional language impairment is due to a specific emotional deficit or to suboptimal general cognitive skills.

Additionally, it is also possible that individuals with ASD with extremely high IQ would also show greater degree of emotional impairment compared to people with average IQ. Other domains, such as restricted and repetitive behaviors, were previously shown to have such complex association with IQ (Bishop et al., 2006). None of the studies included in our review specifically looked at a subgroup of people with ASD with extremely high IQ, although the reported variance of IQ scores suggests that at least a few of their participants had IQ scores in the extremely high range.

So far, the issue of whether emotional language impairments are present to a similar degree in individuals with ASD with varying levels of IQ has not been fully explored. A possible design is to include three groups of subjects with ASD (functioning in the low, middle and superior range of intelligence) and three age and IQ matched non-ASD control groups, and examine whether any discrepancies on emotional language tasks between ASD and matched controls would vary systematically as a function of IQ.

### Are impairments in emotional language related to variation in general intelligence?

Another way to explore the dependance of emotional impairments on IQ differences is to directly calculate the within-group correlation between the performance in the experiment and IQ. If the variation in IQ would matter and would be causally linked to the task performance, we would expect the verbal or nonverbal IQ to negatively correlate with the degree of emotional language impairment within the ASD group. This was examined in seven studies. Three studies found a positive correlation between performance on emotional task and verbal IQ (Gaigg and Bowler, 2009a; Williams and Happé, 2010; Siller et al., 2014), and in three studies the correlation was not significant (Hillier and Allinson, 2002; Lindner and Rosén, 2006; Losh and Capps, 2006). Two studies reported correlations with nonverbal abilities; one study found a significant positive correlation (Hillier and Allinson, 2002), while the other study reported a nonsignificant relationship (Losh and Capps, 2006). Finally, two studies did not look separately at verbal and nonverbal components: one study found a significant positive relationship with mental age (Bauminger, 2004), while the other study found no association with IQ (Han et al., 2014). Because of the small number of studies, it is hard to reach a definite conclusion on whether IQ plays a significant role in performance. However, the fact that only half of the studies found an effect of IQ while the other half found no effect suggests that even if the effect is there, it must be rather weak. Finally, in case of the studies that do not report any data on association between IQ and their measures of interest, it is not clear whether those studies did not investigate the effect of IQ, or whether they found a statistically nonsignificant effect and decided not to report it. A general recommendation for future studies would be to report the relationship between IQ and the outcome of interest, even if this relationship is not statistically significant.

In summary, from these data we can conclude that language delay, presence of intellectual disability or variation in general intelligence cannot fully explain impairments in the processing of emotional language in subjects with ASD. In fact, there is more evidence for impairments of emotion language in high-functioning subjects with ASD and in subjects with Asperger, when compared to appropriate matched control subjects, than in low-functioning subjects with ASD. Nonetheless, variation in general intelligence still plays a role to some degree, although not in all types of tasks, and not with all types of stimuli. The extent to which IQ differences influence emotional language processing remains an open issue that merits further study.

## Consequences of emotional language impairments for our understanding of ASD

So far we have concluded that emotional language impairments in ASD are widespread and not restricted to any particular cognitive domain or stimulus type; they are even more pronounced in subjects with high-functioning autism and Asperger syndrome than in individuals with intellectual disability, language problems or language delay; and they persist through all age groups. These emotional language impairments complement the emotional impairments for the recognition, processing, understanding and production of facial and visual displays of emotions, which have been reviewed extensively by others (Jemel et al., 2006; Harms et al., 2010). In this next section, we will discuss whether and how these widespread and rather general emotional impairments can be accounted for by existing cognitive theories of autism.

### Emotions and global vs. local processing

The central coherence account proposes that autism is characterized by a particular cognitive style of information processing, which can be described as detail-oriented, with particular attention paid to separate parts or aspects of an object (Happé, 2005; Happé and Frith, 2006). In contrast, typical individuals have a strong preference for global processing and integration, also known as central coherence. The evidence supporting the deficit in central coherence in ASD comes from a wide range of studies, including different kinds of visual and semantic tasks (Happé, 2005). However, this theory was developed primarily to explain the pattern of cognitive strengths and weaknesses in autism, and it was not intended to explain the emotional and social-communicative impairments of subjects with ASD.

Weak central coherence seems to explain only part of the evidence with regard to emotional impairments reviewed above. The emotional meaning of an image, a musical piece, or a text may not be evident from the details and only apparent when the stimulus is processed as a whole and/or put into context. If individuals with ASD indeed would miss the emotional meaning of a stimulus because of weak central coherence skills, this would be evident in their explicit evaluation of the stimuli. However, this is not the case; in fact, several studies demonstrated that subjects with ASD could correctly classify spoken sentences as emotionally positive or negative (Volden and Sorenson, 2009; Grossman et al., 2010; Kuchinke et al., 2011), and also provided similar rating of emotional arousal of emotion words as the control group (Corden et al., 2008; Gaigg and Bowler, 2008). Additionally, according to the central coherence account, more complex stimuli (text, stories) would be problematic to subjects with ASD while more simple stimuli such as single words would not be. However, a number of studies found group differences when using single words (Corden et al., 2008; Gaigg and Bowler, 2008, 2009a,b). Finally, if individuals with ASD would be unable to quickly grasp the emotional meaning of a stimulus, we would expect that the neural or autonomic responses to emotional stimuli would not differ from neutral stimuli. However, several studies found that startle potentiation in ASD subjects differs not between positive and negative stimuli, but between these and neutral stimuli, which means that the emotional stimuli do have an effect, but are processed in an atypical way (Wilbarger et al., 2009; Dichter et al., 2010).

There have also been attempts to integrate social-communicative impairments into the central coherence account, by arguing that integrative and holistic processing is essential for social cognition and Theory of Mind skills (Happé, 1997; Jarrold et al., 2000). However, some studies reported that individuals with ASD who pass the Theory of Mind task and those who fail have a similar lack of central coherence performance. This supports the notion that central coherence and theory of mind deficits are independent (Happé, 1997, 2000). Additionally, it is still debated whether central coherence in ASD is weak or deficient, or just unused, because under some task conditions subjects with ASD are able to display holistic processing (Happé and Frith, 2006).

An alternative to the Weak Central Coherence account is the Enhanced Perceptual Functioning (EPF) model (Mottron and Burack, 2001; Mottron et al., 2006). This model proposes that individuals with ASD are not impaired at global processing, but they are better at processing details. As a consequence, spontaneously they choose a more detail-oriented processing style, even though they are able to focus on the global picture when they are explicitly instructed to do so. According to the EPF model, focusing on the perceptual aspects and ignoring the conceptual aspects of the stimuli could lead to a relative lack of processing emotional information. This would explain why participants with ASD would utilize emotional information in some tasks but not in others (Gaigg and Bowler, 2008; South et al., 2008). Future studies need to address this issue, for example, by considering whether a focus on different levels of processing (e.g., superficial perceptual feature analysis or deep semantic analysis) may explain the difference in processing emotion by ASD and typical groups.

### Emotions and executive dysfunction

Another influential theory proposes that the executive dysfunction is at the core of ASD (Ozonoff et al., 2005). Executive function is a broad term that refers to a group of cognitive functions that include monitoring of one’s own behavior, cognitive control, flexibility, inhibition, planning and working memory (Eslinger, 1996). Deficits in various aspects of executive function are observed not only in ASD, but also in many other psychiatric disorders, such as ADHD (Willcutt et al., 2005). Problems in executive function in ASD are especially evident for cognitive flexibility, with inhibitory control and working memory being less affected (Ozonoff et al., 2005).

Research has suggested a link between executive functioning and the processing of emotions (Pessoa, 2009). Research in other clinical populations found that executive control measures correlate with performance in tasks tapping into emotional and motivational systems in schizophrenia (Lee et al., 2009), measures of cognitive control correlate with mood in patients with traumatic brain injury (McDonald et al., 2010), and cognitive flexibility and response inhibition correlate with empathy measures in depressed patients (Thoma et al., 2011). However, emotions and executive functions are thought to be rather separate systems that rely on different brain networks (Seeley et al., 2007). Furthermore, while mood state and emotional significance of the stimuli can certainly have an effect on executive function (Pessoa, 2009; Mueller, 2011), there is no evidence that executive functioning by itself has major effects on emotion processing, in particular on fast stimulus-driven responses (Corden et al., 2008; Gaigg and Bowler, 2009a) and autonomic activity (Kuchinke et al., 2011). Therefore, deficits in executive functioning are not a sufficient explanation for the emotional impairments in ASD.

### Emotions and theory of mind

The development of emotion understanding is tightly linked to the development of social relationships and the accumulation of experience in interaction with others. One of the influential cognitive models of ASD is the Theory of Mind account. It assumes that people normally develop an ability to attribute mental states to themselves and to other people (Leudar et al., 2004; Sodian and Kristen, 2010). This ability is also called Theory of Mind reasoning or mentalizing. Mentalizing is required to reason about thoughts and desires of people, infer their feelings and beliefs, and predict their actions. People with ASD have problems in attributing mental states to themselves or to other people (Yirmiya et al., 1992; Lombardo et al., 2007; Jones et al., 2010; Schulte-Rüther et al., 2011). Reasoning about other people’s emotions is also a part of Theory of Mind abilities. Therefore, problems in deriving emotional meaning from text, describing one’s emotional state, and explaining why one should feel sad or happy might rely on general Theory of Mind ability.

However, differences in emotional awareness in subjects with ASD were found to be independent from impairments in self-reflecting and mentalizing skills (Buitelaar et al., 1999; Silani et al., 2008). Furthermore, while emotions play an important role in social interactions, they are not limited to social phenomena and fulfill important additional psychological functions. For example, emotions play a role in detecting potentially important threatening or pleasant stimuli and triggering an appropriate approach or avoidance behavioral response. Some of the studies dealing with fast automatic responses to motivationally relevant stimuli (angry voice, scary, or pleasant pictures) tapped into this aspect of emotions (Dichter et al., 2010; Kuchinke et al., 2011). Those responses are very fast and bottom-up in nature, and involve a person’s own affective reaction rather than an explicit reflection on his or her own mental state. Those abnormal automatic emotional reactions thus cannot be readily and completely explained by a Theory of Mind deficit.

### Emotions and alexithymia

Recent studies suggest a link between ASD and alexithymia (Silani et al., 2008; Bird et al., 2011). Alexithymia is defined as a difficulty in identifying and describing one’s own feelings and difficulty in distinguishing one’s feelings from bodily sensations of emotional arousal (Nemiah, 1977). Alexithymia is thought to characterize about 10% of the general population (Linden et al., 1995; Salminen et al., 1999). Although alexithymia is neither a necessary nor a sufficient feature of ASD, it is present in approximately 50% of individuals with ASD (Hill et al., 2004; Berthoz and Hill, 2005; Lombardo et al., 2007). Alexithymia is typically assessed based on self-report questionnaires such as TAS-20 (Bagby et al., 1994). The key question here is whether the emotional deficits of subjects with ASD described above are linked to alexithymia (Silani et al., 2008; Bird et al., 2010).

There is evidence that the empathy deficits reported in ASD are indeed related to alexithymia. Subjects with ASD who were not alexithymic demonstrated normal empathic responses, but individuals with ASD and alexithymia showed clear empathy deficits (Bird et al., 2011). The alexithymia account fits with the problems in understanding emotions in text, images, and music. Some studies report that subjects with ASD were able to adequately rate the stimuli on their emotional valence (Corden et al., 2008), which at first glance seems to be contradicting the findings from alexithymia studies. However, the scale used in these studies was rather simple and asked only for a differentiation of positive and negative valence.

Introspection into one’s own inner state is a rather complex skill that only develops in childhood (Flavell et al., 2000), but the first symptoms of autism are often apparent before that. Therefore, it is likely that instead of being a cause, both alexithymia and a lack of empathy in ASD arise as a consequence of impaired emotional processing. For example, some studies report that automatic reflexive responses in ASD do not differentiate between positive and negative valence (Wilbarger et al., 2009; Dichter et al., 2010). These low-level processing abnormalities may give rise to a general problem with identifying and categorizing emotional states in other persons and in oneself. Further studies are needed to examine whether alexithymia is a useful construct to subtype subjects with ASD and to examine the underlying physiological and neural mechanisms of impaired emotion processing and empathy deficits.

### Emotions and motivation: reward and punishment learning

As described earlier, emotions are linked to evaluation of the motivational significance of an object or event and to the motivational system in general (Frijda, 2010; Mesquita and Frijda, 2011). Altered sensitivity to reward would lead to a difficulty in estimating the motivational saliency of the stimuli and hence their emotional meaning. Studies of motivation and processing of reward and punishment in ASD are scarce; however, available data suggest deficits in the processing of reward (Scott-Van Zeeland et al., 2010; Kohls et al., 2011). For example, children with ASD performed abnormally on a delayed non-match to sample task. In this task, a stimulus which is not associated with reward in the current trial will be the one associated with reward in the next trial. Children with ASD had trouble learning stimulus-reward associations and flexibly adjusting them in the course of the task (Dawson et al., 2001). Several neuroimaging studies investigating social (smiling face) and nonsocial (monetary) rewards in subjects with ASD found diminished activations in neural networks associated with processing of reward and punishment: nucleus accumbens (NA; Dichter et al., 2012b; Kohls et al., 2013), ACC (Scott-Van Zeeland et al., 2010; Kohls et al., 2013) and striatum (Scott-Van Zeeland et al., 2010). Additionally, neural response to reward in the left ACC was found to correlate with measures of social interaction abilities in the ASD group (Schmitz et al., 2008).

Another fMRI study used two reward conditions, one consisting of monetary reward, and the other consisting of objects that are typically interesting and relevant for ASD individuals: machines, trains, electronic devices. Control group showed significantly greater activation of the NA in the monetary reward condition, while in the ASD group the NA activity was not significantly different in the two conditions, and not significantly different from the control group in the object reward condition (Dichter et al., 2012a). This result suggests an impairment in the reward processing system in the ASD group, including a diminished anticipation of reward and a lower salience of reward in general. An impairment in reward processing was also found in an ERP study using a different paradigm, where subjects with ASD demonstrated an attenuated P3 component during reward anticipation in response to cues associated with both social and monetary reward (Kohls et al., 2011), suggesting hyporesponsivity to reward in the ASD group and reduced attention allocation to incentive stimuli. However, another EEG study examined a different ERP component (namely, feedback related negativity) and reported no difference between ASD and control groups for positive compared to negative feedback (Larson et al., 2011). This suggests that the dysfunction in the motivational system affects early stages (anticipation of the outcome) to a greater degree compared to later stages (post-feedback processing), and processing of rewards to a greater degree compared to losses.

In all, these data indicate that subjects with ASD are less sensitive to social and nonsocial rewards, as reflected in lower activations of the reward areas of the brain, and even may react with distress to reward given increased activity of the amygdala and insular cortex (Dichter et al., 2012b). The question of whether problems in motivation and sensitivity to reward cause problems in emotions, or, instead, deficient emotional responses lead to problems in motivation, is not fully clear yet. The link between motivational processes and processing of emotional stimuli in ASD needs to be studied further.

### Emotion, simulation, and the mirror neuron system

Embodied simulation theory states that people understand other people’s actions by directly simulating them (instead of logically inferring their intentions through metacognitive reasoning; Gallese, 2007; Gallese and Sinigaglia, 2011). They do so by activating the same brain networks which would also be activated if the observer would be performing the same action. For example, when one observes other people’s actions, a pattern of motor activity is activated that corresponds to the neural pattern of when the observer would perform the same action. According to the simulation theory, this MNS plays an important role in understanding and predicting actions of other people in a rather direct way and without necessarily involving more indirect mentalizing skills. Recently, it has been proposed that our brain simulates not only actions, but also emotions of others (Decety and Jackson, 2004; Keysers and Gazzola, 2006) and in this way facilitates understanding others’ inner emotional states (Perry et al., 2010; Sinigaglia and Sparaci, 2010). Observing other people’s facial expressions is associated with increased activity in regions of the inferior precentral and inferior frontal gyri that are also involved in producing similar expressions (Carr et al., 2003). This activity is thought to trigger congruent activity in emotional brain regions such as the insula and the amygdala (Carr et al., 2003; Jabbi and Keysers, 2008). Activity in the frontal parts of the MNS was found to be associated with the tendency to empathize with other individuals (Gazzola et al., 2006; Jabbi et al., 2007; Saarela et al., 2007; Pfeifer et al., 2008). On the reverse, lesions of this area were associated with deficits in empathy and emotion recognition (Shamay-Tsoory et al., 2009).

A “broken MNS” hypothesis could explain both the social and emotional impairments in ASD (Iacoboni and Dapretto, 2006). This hypothesis is supported by data from behavioral studies and a variety of structural and functional abnormalities found in the MNS region (Dapretto et al., 2005; Hadjikhani et al., 2006). However, recent evidence shows that under different task conditions MNS in autism seems to be intact (Hamilton et al., 2007). Overall, the empirical evidence for the broken mirror hypothesis of ASD is rather mixed and includes a number of studies yielding disconfirming evidence (Hamilton et al., 2007).

It is possible that the abnormal functioning of the MNS observed in the early studies is caused by a deficit outside the MNS. Some authors suggested that the MNS is not dysfunctional on its own, but that motivational abnormalities draw the child’s attention away from social and towards idiosyncratic stimuli. As a result, the MNS does not receive a proper input and therefore does not develop fully (Esser et al., 2010). In a recent review, it was further suggested that other neural systems besides MNS, such as networks involved in attention and control, may be responsible for the inconsistent findings with regard to imitation in ASD (Kana et al., 2011).

## Conclusions and perspectives

The results of our systematic review allow us to draw several conclusions. First, the data supports the existence of impairments in the processing of emotional language in individuals with ASD that are relatively independent of the complexity of stimuli (single words, sentences or discourse), task (production or comprehension, recognition, recall or detection), and of the sensory modality (visual or auditory). These impairments were found in different tasks tapping in reasoning, memory, or attention to emotional stimuli. The evidence for atypical physiological or neural responses is limited due to a small number of studies.

Second, these emotional impairments have been documented in ASD participants with average or even above average cognitive abilities and appear to be rather independent of the level of language development. Studies in low-functioning subjects with ASD, however, are less well suited to differentiate specific problems in handling emotional stimuli from more general emotional deficits due to cognitive problems.

Third, the existence of impairments in emotional language processing has implications for our understanding of the relationship between emotional and social deficits in ASD. In contrast to more concrete stimuli such as faces, linguistic representations of emotion are more abstract, and proficient understanding of them is dependent on verbal ability and develops later compared to reading expressions from faces or body postures (McClure, 2000; Adolphs, 2002). Emotional language can be used in a context that does not involve interaction with another persons. A person could know a word for a specific emotion without having a good understanding of this emotion. For example, people with amygdala lesion become impaired in the ability to understand and recognize fear (Broks et al., 1998), but they do not forget the word “fear” itself. Conversely, it’s also possible to understand another person’s inner state without having a verbal label to describe this state. It seems reasonable to hypothesize that a common emotional impairment may underlie the impairments in both facial emotion processing and in emotional language.

Fourth, we explored how well cognitive accounts of ASD may explain the emotional impairments in ASD. Classic cognitive theories of ASD, namely Theory of Mind, Executive Dysfunction and Weak Central Coherence, have limited power to explain emotional impairments in ASD. On the other hand, findings from studies on reward and punishment learning offer a more promising account of the emotional impairments. More work in this area is warranted. Finally, alexithymia appears to be a useful construct to subtype subjects with ASD, to examine its underlying physiological and neural mechanisms and to link it to broader aspects of emotional functioning in social interaction.

On a broader level, this review indicates that emotional impairments in ASD should receive more attention from both researchers and clinicians. Emotional impairments in ASD were brought forward by Kanner already in his seminal description in 1943, but since then have shifted to the background. In the current classification systems DSM-5 and ICD-10, emotional symptoms are regarded as part of, or as secondary to, problems in social interaction and communication. Bringing emotional symptoms in ASD to the attention of researchers may inform our understanding of ASD in several ways. We can then establish whether emotional impairments are present across the whole autism spectrum, are specific for ASD, or resemble the deficits observed in other disorders such as schizophrenia or anxiety disorders. Further, this will allow us to provide a more detailed and accurate description of the clinical manifestations of ASD. This would facilitate identification of more homogeneous subgroups and stimulate the study of emotional competence of subjects with ASD within and outside the social context and across various levels of structure of social contexts. Finally, this would increase investments in developing and testing therapeutic interventions for emotional deficits in ASD.

Emotional and social development are tightly linked to each other. Many emotions, such as shame or embarrassment, are social in nature, and displays of emotion are important elements of everyday social interactions. Trouble understanding the emotions of other people can lead to great difficulties in social functioning. The emotional aspects of social interaction received considerable attention from researchers (Begeer et al., 2008). However, ‘emotional’ and ‘social’ are not the same. Emotions do not only have social, but also psychological and biological components, are tightly related to the system of motivations and needs (Frijda, 2008; Larsen et al., 2008; Mesquita and Frijda, 2011), and are responses to unconditional stimuli such as pain. In turn, social interaction cannot be reduced to purely emotional aspects. Various models of social interaction include cognitive components, such as overt encoding and interpretation of behavioral cues and selecting an appropriate response (Crick and Dodge, 1994, 1996), and implicit cognition such as implicit judgment biases and memory (Greenwald and Banaji, 1995; Amodio and Ratner, 2011). Some authors suggest that simulation is a crucial component of social cognition (Barsalou, 2008), and that the MNS is the neural mechanism of simulation (Wolpert et al., 2003; Uddin et al., 2007). Proponents of this view claim that the motor system is essential for social cognition (Gallese, 2007).

Social and emotional development are intertwined; social interactions with other people and observation of their emotional reactions are of the utmost importance for the development and maturation of emotional competence. An impoverished pattern of social experiences as in ASD will have its consequences for the emotional development of an individual.

Are the observed emotional deficits in subjects with ASD only a consequence of limited normal social experiences, or are they present from the very start and do they contribute to the abnormalities in social interaction? Recent reviews in this field have already suggested that impairments of emotion processing in ASD should not be viewed as a mere consequence of core social deficits, but instead as a result of an interplay between emotion and various cognitive processes as well as social development (Gaigg, 2012; Nuske et al., 2013). The data on emotional deficits in adolescents and adults with ASD indicate that these are not limited to the context of social interaction, but also found in tasks that do not directly rely on social interaction or communication skills, such as memory and attention tasks. However, there is still a possibility that these emotional deficits have been ultimately caused by social deficits that affect the development of the emotional competence and emotion understanding: Because a child with ASD is not able to establish normal social contact, his/her knowledge and experience related to emotions are also limited. One way to clarify this issue is to investigate the development of reciprocal social interaction and the amount of social experience along with the development of emotional competence using a longitudinal design. Such a study design would allow a disentanglement of the mutual influence of social and emotional development. Unfortunately, so far not enough developmental studies are available, and the developmental course of this impairment needs further study. Another approach is to compare the processing of emotion in language to other language aspects that would equally rely on social interaction and social understanding, and to test if emotional language processing is impaired above and beyond general semantic or pragmatic language processing. The stimuli in the studies reviewed here are difficult to separate into social vs. nonsocial types. One can rather speak about the degree to which a task has a social component. For example, having a structured interview or having to explain the meaning of a word to the experimenter can be viewed as a more social task, while listening to recorded speech is less social, and hearing or reading word lists is even less social. None of the studies included in this review manipulated social and emotional aspects of the task independently, although several studies attempted to compare social and emotional aspects of the stimuli (Hobson and Lee, 1989; Beversdorf et al., 1998).

The consideration of social and emotional functioning as rather distinct but closely interacting domains during development has implications for designing and performing longitudinal studies of subjects with ASD. These studies should assess both social and emotional functioning and examine whether and how social problems aggravate problems of emotional functioning, and vice versa in healthy and ASD subjects. Furthermore, research should address whether subjects with ASD try to compensate for their deficit in the processing of social or emotional stimuli by using learned cognitive strategies. In addition, it is important to disentangle the contributions of specific cognitive functions to the problems in the emotional domain and in the social domain to better understand the nature of the disorder and to develop appropriate intervention strategies.

There is also an urgent need for translational cognitive and neuroscience studies in ASD. These should bridge the gap between sophisticated research into cognitive and neural mechanisms in selected and rather small samples of ASD subjects, and observing and analyzing social and emotional functioning in daily life in larger and more representative groups of subjects with ASD. The final test of prospective cognitive and neural markers of ASD is their potential to improve our understanding of day-to-day functioning and adaptation, and to form a basis for clinical intervention and management.

## Conflict of interest statement

The authors declare that the research was conducted in the absence of any commercial or financial relationships that could be construed as a potential conflict of interest.

## Abbreviations

ABC: Autism Behavior Checklist
ADI: Autism Diagnostic Interview
ADOS: Autism Diagnostic Observation Schedule
AS: Asperger syndrome
ASD: Autism Spectrum Disorder
ASDS: Asperger Syndrome Diagnostic Scale
CA: chronological age
CARS: Childhood Autism Rating Scale
DD: developmental delay
DS: Down syndrome
LFA: low-functioning autism
MA: mental age
NVIQ: nonverbal intelligence quotient
NVMA: nonverbal mental age
PDD: Pervasive Developmental Disorder
PIQ: Performance intelligence quotient
TD: typically developing
VIQ: verbal intelligence quotient
VMA: verbal mental age
WS: Williams syndrome.
